# The two faces of JAK-STAT

**DOI:** 10.7554/eLife.112188

**Published:** 2026-07-16

**Authors:** Qianying Lu, Yingyi Zhang

**Affiliations:** 1 School of Disaster and Emergency Medicine, Tianjin University Tianjin China; 2 Tianjin Key Laboratory of Disaster Medicine Technology Tianjin China

**Keywords:** immunotherapy response, Breast tumors, JAK-STAT pathway, anti-PD-1 therapy, T cells, two-sided signals, Human

## Abstract

A signal that can help breast cancer cells grow may also increase immune responses and boost immune therapy.

**Related research article** Zhou J, Zhang H, Tang H, Yu L, Peng F. 2026. JAK-STAT pathway heterogeneity governs immunotherapy response in breast cancer. *eLife* **15**:RP110635. doi: 10.7554/eLife.110635.

Breast tumors are not made of cancer cells alone. They are more like busy neighborhoods, where cancer cells, immune cells, blood vessels and supporting cells exchange signals. Some signals help the tumor grow, while others help the immune system fight back. The same signal can thus mean different things in different cells.

One example is the JAK-STAT pathway. It helps cells respond to messages from their surroundings. Signals at the cell surface activate JAK proteins, which then activate STAT proteins. STAT proteins enter the nucleus and switch genes on or off. This pathway can help immune cells become active. But in cancer, this pathway can also support tumor growth.

This double role has made breast cancer biology more complex. A type of cancer treatment known as anti-PD-1 therapy, which helps the immune system recognize and attack cancer cells, can improve outcomes for some patients with triple-negative breast cancer by blocking PD-1, an inhibitory receptor that restrains T-cell activity (Schmid et al., 2020; Cortes et al., 2022). This treatment does not directly decrease JAK-STAT; instead, JAK-STAT activity may increase as T cells become more active. Indeed, the activity of the JAK-STAT pathway has been shown to increase after this therapy and has been linked to better treatment responses (Voorwerk et al., 2019; Bassez et al., 2021). However, this seems contradictory, since the JAK-STAT pathway also furthers tumor growth. Why, then, would an increased pathway activity mean better responses to anti-PD-1 treatment?

Now, in eLife, Jianbo Zhou, Heng Zhang, Hailin Tang, Lei Yu and Fu Peng offer a simple answer (Zhou et al., 2026). The key question to ask is not only how strong the JAK-STAT signal is, but which cells are using it. The researchers, based at various research institutes in China, combined single-cell RNA sequencing, spatial transcriptomics, anti-PD-1 patient data, and cell experiments to study the distinct cell states present within the same breast cancer tissue.

The first finding was clear. JAK-STAT activity was not evenly distributed across the tumor. It was strongest in immune cells, especially T cells, which can recognize and kill abnormal cells. Many tumor cells and healthy epithelial cells showed lower JAK-STAT activity. As a result, measuring JAK-STAT activity across an entire tumor can mask these cell-type-specific differences because the signal represents an average of all cells rather than the distinct activity within individual cell populations.

In tumor cells, high JAK-STAT activity was generally lower than in T cells. However, within the tumor-cell population, higher activity still marked harmful behavior. These cancer cells showed stronger signs of growth, invasion and survival. They also showed more MAPK and NF-kB activity, two major signaling paths involved in tumor growth and progression. Zhou et al. further found evidence for MIF-CD74 signaling, a pathway that may create an immune-suppressive environment. In other words, JAK-STAT signaling inside tumor cells may help cancer cells grow and avoid immune attack by activating other pathways involved in tumor growth.

In T cells, however, a high JAK-STAT activity was associated with an improved immune response. The cells expressed more genes involved in killing tumor cells and stronger interferon responses, which help the body detect abnormal cells. These T cells also expressed fewer genes linked to T-cell exhaustion, a state in which T cells gradually lose their ability to attack after long or repeated stimulation, suggesting that they had not lost their ability to fight.

The findings of Zhou et al. help explain why patients with higher overall JAK-STAT activity may respond better to immunotherapy. In a standard tumor sample, signals from many cell types are mixed. A high score may reflect active T cells, as well as aggressive cancer cells. In line with this, Zhou et al. found that patients with higher pathway activity were more likely to respond to anti-PD-1 therapy, especially in triple-negative breast cancer, a subtype with fewer treatment options (Wolf et al., 2022).

Zhou et al. further focused on STAT4, a member of the STAT family. Like the signaling pathway, it was linked to a stronger immune response in T cells. This is also consistent with recent work showing that IL-12, an immune signal that activates JAK-STAT signaling, can drive lymphocytes towards a prolonged, tumor-killing state (Landoni et al., 2024). In breast cancer cells, increasing STAT4 promoted tumor growth and formation. STAT4 also increased the expression of SLC47A1, a gene that may help cancer cells avoid a form of cell death known as ferroptosis (Lin et al., 2022).

These findings advise against treating JAK-STAT signaling as simply good or bad. Broadly blocking the pathway may reduce tumor-promoting signals in cancer cells. But it could also weaken useful T cell responses or disturb normal epithelial cells, which rely on regulated signaling programs to maintain tissue states (Pal et al., 2021).

A better strategy may be to consider cell type, timing and breast cancer subtype. Measuring JAK-STAT activity in T cells could help identify patients who may benefit from anti-PD-1 therapy. Blocking tumor-intrinsic JAK-STAT signaling, or the MIF-CD74 pathway, might improve responses. Future work should ask how to measure this pathway, what to target, and when such drugs should be given. The message is simple: the meaning of JAK-STAT signaling depends on who is listening.
